# Computer-Aided Virtual Screening and In Vitro Validation of Biomimetic Tyrosinase Inhibitory Peptides from Abalone Peptidome

**DOI:** 10.3390/ijms24043154

**Published:** 2023-02-05

**Authors:** Sasikarn Kongsompong, Teerasak E-kobon, Weerasak Taengphan, Mattanun Sangkhawasi, Mattaka Khongkow, Pramote Chumnanpuen

**Affiliations:** 1 Interdisciplinary Graduate Program in Bioscience, Faculty of Science, Kasetsart University, Bangkok 10900, Thailand; 2 Department of Genetics, Faculty of Science, Kasetsart University, Bangkok 10900, Thailand; 3 Omics Center for Agriculture, Bioresources, Food and Health, Kasetsart University (OmiKU), Bangkok 10900, Thailand; 4 Expert Centre of Innovative Herbal Products (InnoHerb), Thailand Institute of Scientific and Technological Research, Techno Polis, Khlong Luang District, Pathum Thani 12120, Thailand; 5 Program in Biotechnology, Faculty of Science, Chulalongkorn University, Bangkok 10330, Thailand; 6 National Nanotechnology Center (NANOTEC), National Science and Technology Development Agency (NSTDA), Pathum Thani 12120, Thailand; 7 Department of Zoology, Faculty of Science, Kasetsart University, Bangkok 10900, Thailand

**Keywords:** tyrosinase, peptide, inhibitor, bioinformatics, abalone, biomimetics

## Abstract

Hyperpigmentation is a medical and cosmetic problem caused by an excess accumulation of melanin or the overexpression of the enzyme tyrosinase, leading to several skin disorders, i.e., freckles, melasma, and skin cancer. Tyrosinase is a key enzyme in melanogenesis and thus a target for reducing melanin production. Although abalone is a good source of bioactive peptides that have been used for several properties including depigmentation, the available information on the anti-tyrosinase property of abalone peptides remains insufficient. This study investigated the anti-tyrosinase properties of *Haliotis diversicolor* tyrosinase inhibitory peptides (hdTIPs) based on mushroom tyrosinase, cellular tyrosinase, and melanin content assays. The binding conformation between peptides and tyrosinase was also examined by molecular docking and dynamics study. KNN1 showed a high potent inhibitory effect on mushroom tyrosinase with an *IC*_50_ of 70.83 μM. Moreover, our selected hdTIPs could inhibit melanin production through the reductions in tyrosinase activity and reactive oxygen species (ROS) levels by enhancing the antioxidative enzymes. RF1 showed the highest activity on both cellular tyrosinase inhibition and ROS reduction. leading to the lower melanin content in B16F10 murine melanoma cells. Accordingly, it can be assumed that our selected peptides exhibited high potential in medical cosmetology applications.

## 1. Introduction

Melanin is the primary pigment found in human hair, eyes, and skin, and is produced by melanocytes. The melanocytes produce two types of melanin: eumelanin (brownish black) and pheomelanin (reddish yellow) [1,2]. Melanin has an important role in UV-protection, especially of human skin. However, excess accumulation of melanin causes dermatological problems such as freckles, lentigo, post-inflammatory, melasma, as well as skin cancer [3,4,5,6].

Tyrosinase, a key enzyme in the melanin production process, is mainly produced by melanocyte cells. This enzyme has two copper ions surrounded by three histidine residues that respond to catalytic activity [7]. Tyrosinase catalyzes a two-step oxidative reaction: the hydroxylation of L-tyrosine to L-3,4-dihydroxyphenylalanine (L-DOPA), followed by the oxidation of L-DOPA to L-dopaquinone. These steps are named monophenolase and diphenolase activities, respectively. The L-dopaquinone from the diphenolase reaction is spontaneously converted to dopachrome, which will be further transformed into melanin through a series of non-enzymatic processes [8,9]. Tyrosinase activity can be assessed based on the formation of dopachrome, which is a brown compound with a peak absorbance at 475 nm that is detectable using a spectrometer [10,11].

Due to the importance of tyrosinase in melanin synthesis, the inhibition of tyrosinase is the main target of treating hyperpigmentation [12]. Among the various tyrosinase sources, mushroom tyrosinase from *Agaricus bisporus* is a low-cost source with commercial availability, high similarity, and homology compared to human tyrosinase [13]. It is widely used as an enzymatic in vitro model for developing the skin lightening agents [7]. Tyrosinase inhibitors have been used as therapeutic agents for skin pigment disorders. Several well-known inhibitors, such as kojic acid (KA), hydroquinone (HQ), and arbutin, have been used worldwide under restrictions to avoid their side-effects [14,15,16]. KA, HQ, and arbutin were reported to have low cell penetration ability and also cause contact dermatitis and erythema after long-term use [17,18,19]. KA also has low stability during long-term storage and insufficient tyrosinase inhibition activity [20]. Therefore, novel tyrosinase inhibitors with high potential but low side-effects are urgently required. According to these perspectives, tyrosinase inhibitory peptides (TIPs) are promising alternatives to natural tyrosinase inhibitors. Beside the direct inhibitory effect on tyrosinase enzyme, TIPs have also been reported to inhibit melanin production by reducing the reactive oxygen species (ROS). The mechanisms of TIPs on ROS reduction could either be the direct effect by their antioxidative activity or the indirect effect through the activation of intracellular antioxidative enzymes. These key enzymes, i.e., super-oxide dismutase (SOD), catalase (CAT), and glutathione peroxidase (GPx), could be the possible activation targets for TIPs [21,22,23].

Currently, consumers are becoming more aware of the nutritional value of food in order to boost health, lower the frequency of diseases, and lengthen life spans through the consumption of dietary nutrients [24]. Abalone is considered as a delicacy with high commercial value compared to other shellfishes in Asian countries and regarded as the “ginseng of the ocean” with several health-promoting effects through nutrients and bioactive components [25,26]. Abalone meat and their product processing wastes are good-quality protein resources since the protein content of abalone muscle, viscera, and gonad was notably high (approximately 50% and 25% of dry weight in muscle and gonad/viscera, respectively) [27]. Along with the high consumption rate of abalone meat. the abandonment of processing waste is more than 27,000 tons (based on 15% of discard rate) of viscera and gonad tissue every year [28]. Therefore, abalone protein and peptide are becoming a very promising candidate for dietary nutrients and skin care products [25].

Abalone peptides are proteins that have been hydrolyzed into smaller units that are capable of several nutritional functions and biological activities [29]. These peptides are derived from the muscle tissue, mantle, and visceral organs of the abalone [30,31]. Abalone peptides are believed to have a number of potential health benefits, including anti-inflammatory and antioxidant effects, and may be used in supplements and other health-related products [29,30,31,32,33,34]. Some studies have also suggested that abalone peptides have several potential bioactivities such as anti-tumor effects, immune function improvement, anti-microbial, anti-thrombotic, anti-coagulant, and angiotensin-I-converting enzyme (ACE-1) inhibitory activities [32,33,35,36,37,38,39]. For cosmetic purposes, abalone peptides also have a high potential for skin-whitening or lightening effects, as well as anti-photoaging effects, due to their antioxidant, anti-inflammatory, reactive oxygen species (ROS) reducing, and matrix metalloproteinases (MMPs) inhibition properties [40,41]. However, the anti-tyrosinase activity of the abalone peptides has never been investigated before. According to our previous study on the tyrosinase inhibitory peptides (TIPs) initially predicted using machine learning-based bioinformatic approaches [42], TIP candidates from the abalone peptidome have been selected for molecular docking analysis and antioxidative prediction to gain more computational evidence of their potential anti-tyrosinase activity. This computer-aided analytic platform helped select a smaller list of the abalone peptide candidates, called *Haliotis diversicolor* tyrosinase inhibitory peptides (hdTIPs), for chemical synthesis and further in vitro analysis by measuring melanin content, mushroom and cellular tyrosinase activities, ROS level, and antioxidative enzyme activities. Results of this study have suggested another useful property for adding more values to the abalone peptides from the global abalone farming and processing industry. The focus on this peptidome allowed implementation of extensive computational analysis and experimental validating of specifically-targeted peptides, and would benefit further exploration of other organismal peptidomes.

## 2. Results

### 2.1. Tyrosinase Inhibitory Peptide Candidates

Previously, our research team proposed the in-house machine learning-based anti-tyrosinase prediction tool, which provides thousands of potential peptides. The probability scores of all 1079 peptides from KNN-based and RF-based models can be found in the supplementary file of the previous article by Kongsompong et al. [42]. The KNN and RF predictors were trained and tested against 133 peptides with known anti-tyrosinase properties with 97% and 99% accuracy [42]. The predictions of both models were based on the numeric matrix of 425 features calculated from amino acid composition (20 features), di-amino acid composition (20 × 20 features), and 5 physicochemical property features (hydrophobicity, peptide length and mass, and numbers of positive charge and negative charge residues). The KNN predictor suggested 1075 putative TIPs and six TIPs from the RF predictor (Table S1). The selected top-eight peptide candidates from the TIP predictor have been classified by prediction probability score as follows (Table 1). TIP1 and TIP2 were then positive predicted TIPs (with over 0.5 probability scores) by both TIPs predictors, while KNN1-3 and RF1-3 are the top-three predicted TIPs by either KNN or RF predictors, respectively. The predictive antioxidant scores calculated by the AnOxPePred-1.0 program were also shown for putative free radical scavenging and ion chelating activities.

Only TIP1 and 2 showed the predictive probability greater than 0.5 on both TIPs predictors (KNN- and RF-based machine learning models). Contrary, KNN1-3 and RF1-3 were the top-three highest scored TIPs from KNN- and RF-base models, respectively. According to the AnOxPePred scores, TIP2 and KNN1 were putative TIPs with the highest predictive antioxidative scores in free radical scavenging and ion chelating properties, respectively. Notably, TIP1-2, KNN2, and RF1 were ranked as top two for ion chelating ability.

### 2.2. Cytotoxicity of hdTIPs on B16F10 Cell Line

**Figure 1:**
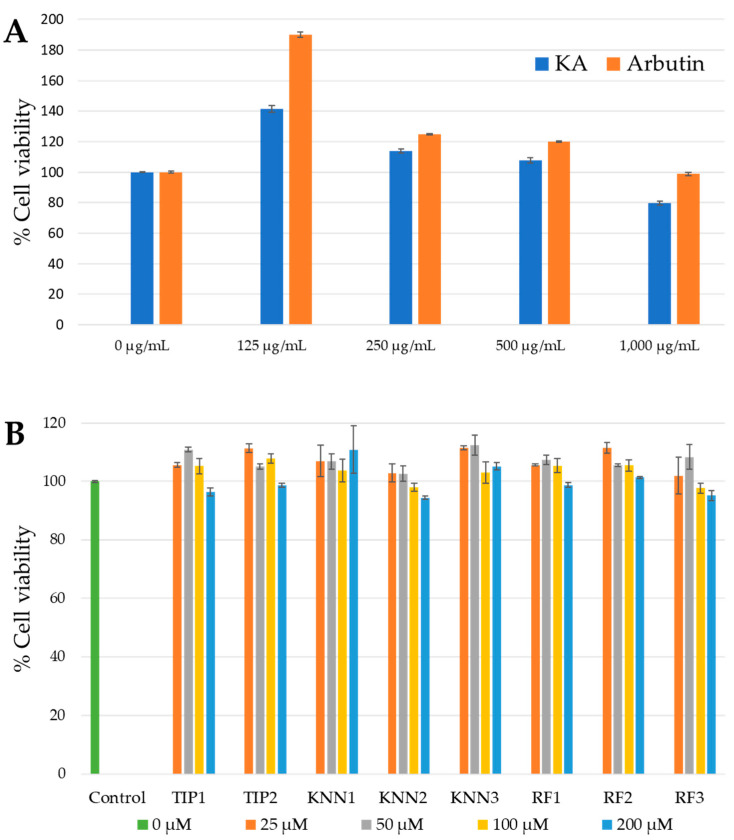
Effect of positive controls (kojic acid and arbutin) (**A**) and hdTIPs (**B**) on B16F10 melanoma cell viability, as assayed by MTT. The data were normalized by setting 100% equal to the viability of the untreated control group. Error bars indicate the standard deviation of mean among the three replications in each treatment groups.

To evaluate the probable effects of each hdTIPs candidate on melanoma cells, the standard MTT assay was carried out to investigate the survival rate of B16F10 melanoma cells after exposure to hdTIPs at five concentrations (0, 25, 50, 100, and 200 μM). Cells exhibited a survival rate of over 90% up to concentrations of 200 μM (Figure 1) after a 24-h incubation period. To also ensure the cytotoxic effect of both positive controls, two commercial whitening agents, kojic acid and arbutin at 0–1000 μg/mL, were also tested on the melanoma cell line (Figure 1A). At maximum concentration, only kojic acid showed approximately 20% of cell proliferation inhibition effect on the melanoma cell. The results showed that the tested concentrations of all hdTIPs and arbutin (another positive control) did not affect the cell proliferation of melanoma cells (Figure 1B). Based on the statistical analysis using Student’s *t*-test calculation, there were no significant differences in survival rates between the treated and control groups. According to this MTT assay results, the concentration of both positive controls was set at 100 μg/mL for the rest of the experiments to avoid the cytotoxic effect on B16F10 cell lines.

### 2.3. Inhibitory Effect of hdTIPs on Mushroom Tyrosinase 

**Figure 2:**
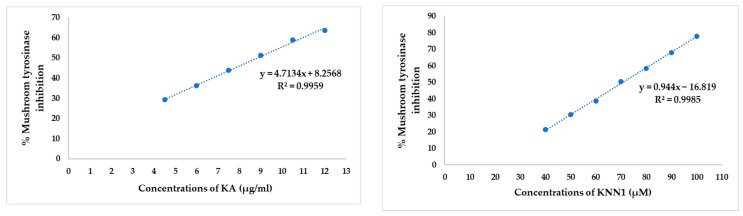
The inhibitory effect of kojic acid (**A**) and hdTIP (KNN1) (**B**) against diphenolase activity of mushroom tyrosinase.

To determine whether the peptides directly affect on tyrosinase activity, mushroom tyrosinase assay was performed using L-DOPA as a substrate. KNN1, the only one of the eight hdTIPs, exhibited the mushroom tyrosinase inhibitory property with an *IC*_50_ of 70.83 μM (Figure 2). The other peptides, on the other hand, showed no inhibitory effect up to a concentration of 500 μM, indicating that these hdTIPs (except KNN1) did not inhibit the diphenolase activity of mushroom tyrosinase. The *IC*_50_ from this experiment has been considered and used for the experimental design in further cellular screening methods.

### 2.4. Inhibitory Effect of hdTIPs on Cellular Tyrosinase and Melanin Content

**Figure 3:**
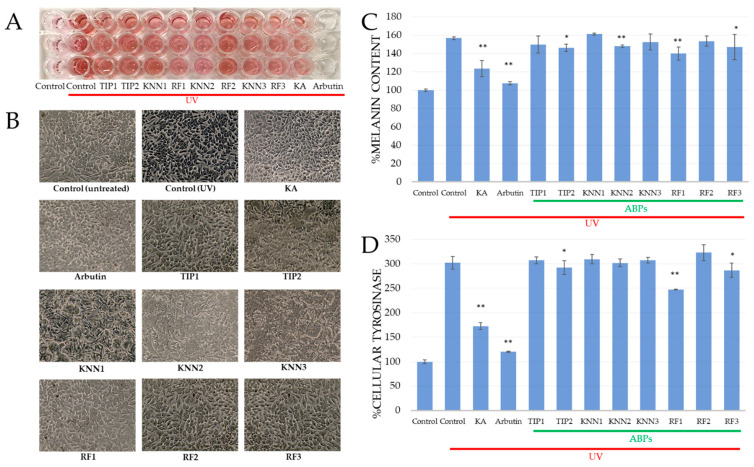
Effects of hdTIPs on melanin content (**A**–**C**) and (**D**) cellular tyrosinase activity of B16F10 melanoma cells. (**A**) Photography of cultured cell with medium in 96-well microplate and (**B**) morphological observation of B16F10 cells treated with hdTIPs. The data were normalized by setting 100% equal to the non-UV-induced of the untreated control group. The significant differences were indicated at *p* < 0.01 (**) and *p* < 0.05 (*). Error bars represented standard error of the mean.

The inhibitory activity of abalone biomimetic peptides against cellular tyrosinase and melanin content was investigated in a melanoma cell line (B16F10). The cells were stimulated by UVA/UVB, then treated with the abalone biomimetic peptides at 70 μM, approximately the same concentration as in the mushroom tyrosinase assay. Both arbutin and kojic acid were used as positive controls. Compared to the non-UV-treated control, the UV-induced cells showed 3-fold higher cellular tyrosinase activity (Figure 3A) and about 1.5-fold higher melanin production (Figure 3B). RF1 represented the most inhibition both in cellular tyrosinase (18.26%) compared to 10 μg/mL kojic acid (42.93%) and in melanin content (10.70%) compared to 100 μg/mL kojic acid (21.08%). Notably, the KNN1 peptide, which can inhibit mushroom tyrosinase, was unable to inhibit either cellular tyrosinase or melanin content at the same concentration. On the other hand, RF3 and TIP2 were in the second (5.11%) and third (3.32%) ranks for inhibition of cellular tyrosinase. Alternately, in terms of melanin content, TIP2 was the second-most-effective inhibitor (6.71%) and RF3 was the third-most-effective inhibitor (6.09%) of melanin production. Furthermore, KNN2 was the fourth-most-effective inhibitor in both assays. The inhibition pattern in cellular tyrosinase was similar to the melanin content, suggesting a strong correlation between the cellular tyrosinase inhibition and the melanin content decrease.

### 2.5. Effects of hdTIPs on ROS Levels and Intracellular Antioxidant Activities

**Figure 4:**
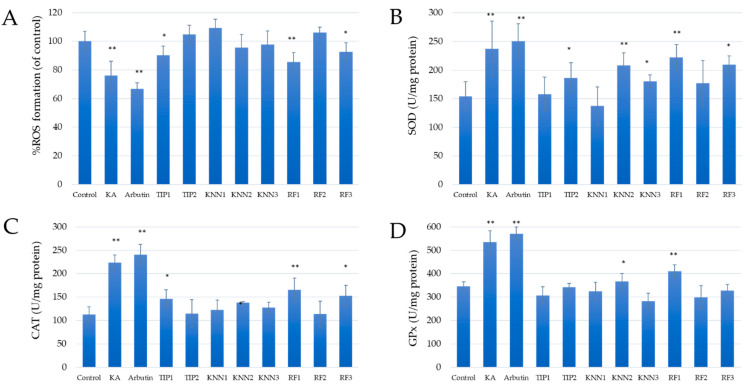
Effects of hdTIPs on reactive oxygen species (ROS) level and antioxidative enzyme activities of melanoma cells. (**A**) Relative percentage of ROS levels compared to untreated control group, (**B**) superoxide dismutase, (**C**) catalase, and (**D**) glutathione peroxidase. The significant differences were indicated at *p* < 0.01 (**) and *p* < 0.05 (*). Error bars represented standard error of the mean.

Tyrosine is necessary for the formation of dopamine in an oxidized environment, which leads to the generation of dopamine in the process of melanin production. Thus, the presence of reactive oxygen species such the hydroxyl radical and superoxide anion facilitates the production of melanin [43]. Thus, the presence of reactive oxygen species such the hydroxyl radical and superoxide anion facilitates the production of melanin [43]. To determine the effect of hdTIPs on ROS level in B16F10 cells and the three main antioxidative enzymes after UV exposure, the enzyme activity assays of 70 μM treatments of each peptide were performed and shown in Figure 4A, where kojic acid (KA) and arbutin (at 100 μg/mL concentration) were used as positive controls. RF1, TIP1, and RF3 showed the significant reductive effects on the relative percentages of ROS in melanoma cells compared to the untreated control group. Interestingly, these ROS levels were positively correlated with the melanin content in Figure 3C. According to the antioxidative enzymes activities result, these three hdTIPs had significant promotive effects on SOD, CAT, and GPx (Figure 4B–D). Notably, KNN2 only elevated SOD and GPx activities, while RF3 only affected SOD and CAT enzymes. These findings suggest that hdTIPs can obliterate the oxidative environment in cells. By regulating the level of ROS in cells and maintaining the reductive capacity in cells, it can stop the synthesis of melanin through the promotive effect on antioxidative enzymes.

### 2.6. Molecular Docking Simulation of Selected hdTIPs on Tyrosinase

**Figure 5:**
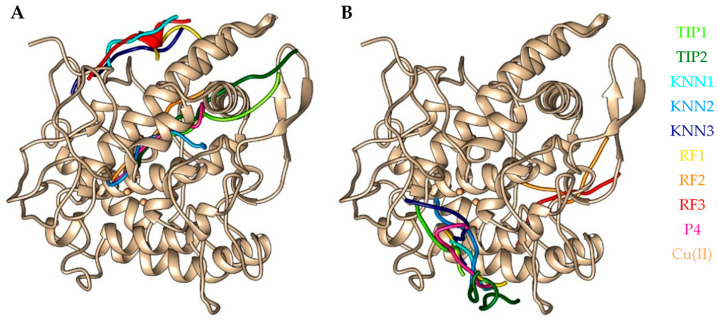
Comparative molecular docking of the hdTIPs and the positive control peptide (P4) on the crystal structure of polyphenol oxidase subunit of tyrosinase (PDB: 2Y9X) from different online protein-peptide docking tools (**A**): GalaxyPepDock and (**B**): HPEPDOCK. The structure of the tyrosinase is shaded in gold, and the peptides are labeled with different colors.

In order to determine whether the abalone biomimetic peptides are able to bind to the active site of the tyrosinase enzyme, the molecular docking of the hdTIPs to the polyphenol oxidase domain (chain A–D) of crystal structure of tyrosinase from *Agaricus bisporus* (PDB: 2Y9X) was performed by two protein-peptide docking tools, GalaxyPepDock (template-based docking program) and HPEPDOCK (global docking program). P4, YRSRKYSSWY, also known as decapeptide-12, was used as a reference peptide for a molecular docking study. Other researchers reported that P4 is the best-known model peptide that has been commercialized and serves as the main active ingredient found in the LumixylTM skin lightening product [44,45]. As shown in Figure 5, the molecular docking results indicated the different docking positions of all selected peptides on protein crystal structure of tyrosinase based on two protein-peptide docking webservers. Figure S1 shows the binding site and hydrogen bond separately for each peptide. Although the binding affinities of the peptides are quite varied, KNN1 showed a higher binging affinity (−9.8 kcal/mol) than P4 (−9.3 kcal/mol) according to the results of HPEPDOCK analysis. In terms of molecular docking score, both KNN1 (−47.9058 kJ/mol) and RF1 (−150.03 kJ/mol) showed a higher level of binding energy compared to P4 (−19.5013 kJ/mol) (Table 2). In addition, Table S3 lists all hydrogen bonds of peptides to tyrosinase proposed by molecular docking simulation.

### 2.7. Molecular Dynamic Simulation of Tyrosinase-hdTIPs Complexes

Since molecular docking and molecular dynamics methods can provide such valuable insights regarding the physicochemical properties of bioactive molecules, they are commonly used as a computer-aided virtual screening strategy. To ensure the binding stability of tyrosinase-hdTIPs complexes, the molecular dynamic simulation of the top selected hdTIPs (KNN1, RF1, RF3, and TIP2) binding to the tyrosinase backbone was performed for 300 nanoseconds to examine the conformational stability and fluctuation analysis of the complex. The stability of the hdTIPs and tyrosinase complex was estimated by RMSD, Rg, and RMSF trajectory analysis.

**Figure 6:**
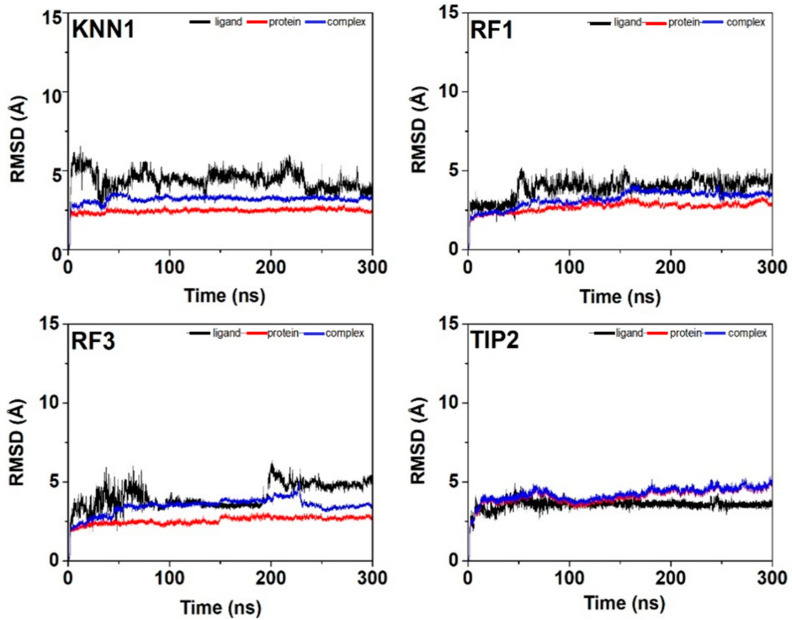
RMSD profiles of the hdTIPs (ligand), tyrosinase protein backbone, and tyrosinase-hdTIPs complexes.

**Figure 7:**
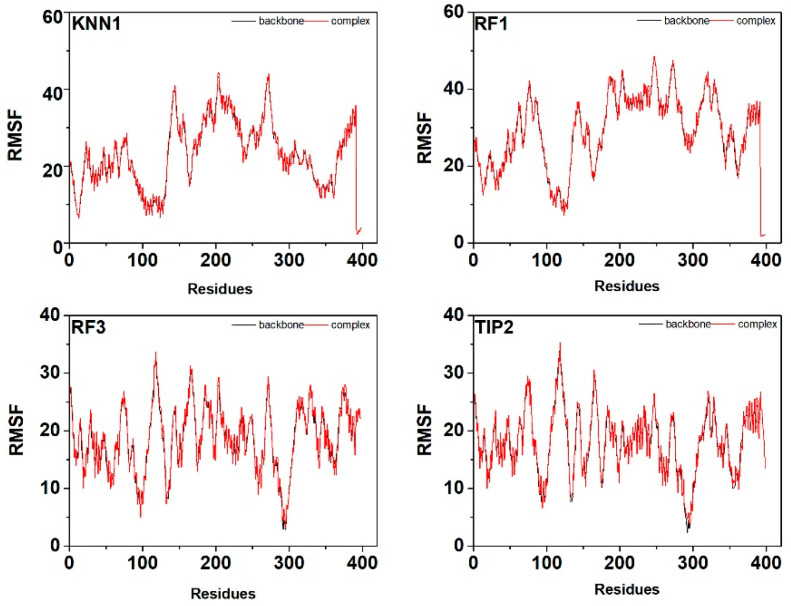
RMSF profiles of the tyrosinase-hdTIPs complexes during 300 ns of the molecular dynamic simulation time.

The low fluctuation pattern of the RMSD profile represents the higher stability of the interested protein-peptide complex [46]. The RMSD values of each ligand, protein backbone, and their complexes remained stable in the range of 3–5 Å. As shown in Figure 6, the tyrosinase complex with hdTIPs was quite rigid with less than 5 Å RMSD, and they had a similar trend for 300 ns of the simulation time as the apo form of the protein backbone for the last period of dynamics. The result indicated that all protein-peptide complexes remained stable after a certain period of time. After that, the RMSF profiles of the tyrosinase-hdTIPs complex were also generated to determine the conformational stability of the protein-peptide complex (Figure 7). The low fluctuation of coordinates in the range of 10–40 Å indicates the high stability of the protein-peptide complexes.

**Figure 8:**
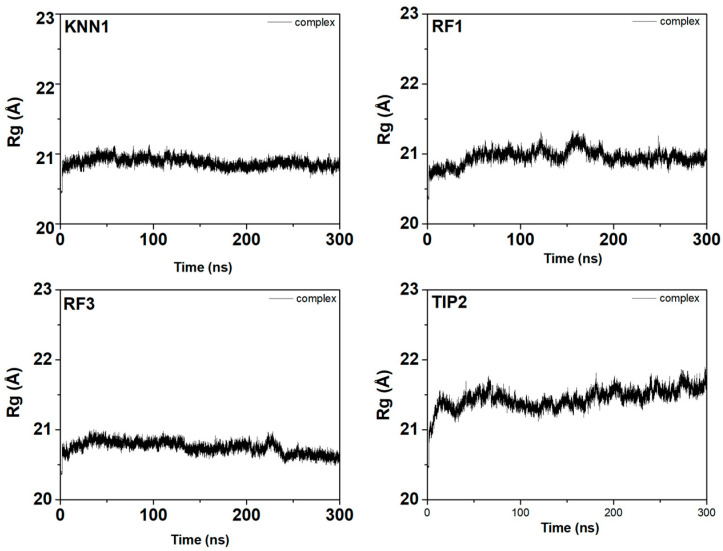
Radius of gyrus plots of the tyrosinase-hdTIPs complexes during 300 ns of the molecular dynamic simulation time.

**Figure 9:**
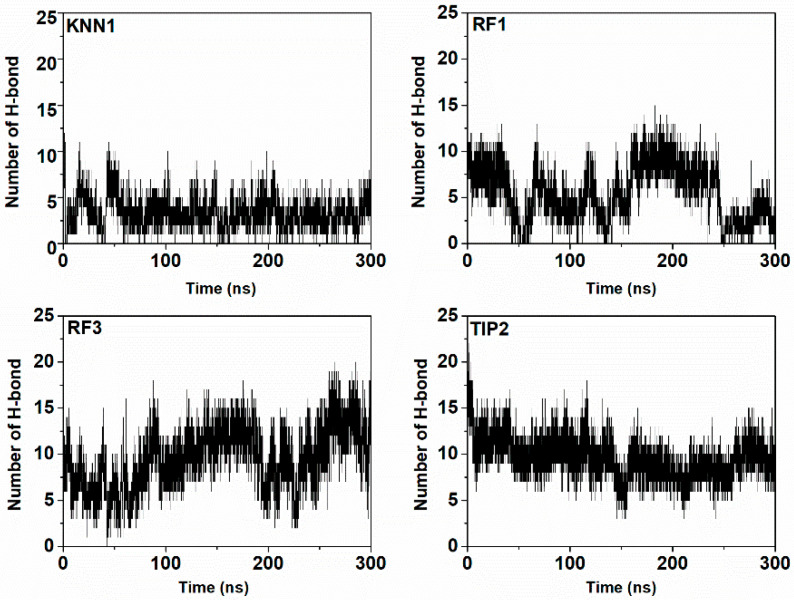
Number of hydrogen bonds plots of the tyrosinase-hdTIPs complexes during 300 ns of the molecular dynamic simulation time.

To define the structural activity of the enzyme-inhibitor complex, the radius of gyration (Rg) of the involved trajectories was also simulated (Figure 8). The Rg value slightly fluctuated according to the folding state of the tyrosinase-hdTIPs complexes. Low fluctuations were observed in the range of 20.5–21.5 Å, indicating the stability of the tyrosinase protein backbone during the binding with each peptide candidate. Finally, hydrogen bond involvements were investigated in order to calculate the dynamic equilibration of the tyrosinase-hdTIPs complexes. The hydrogen bonding profile with a high number of hydrogen bonds during the simulation period indicated the stable binding of hdTIPs with the target tyrosinase enzyme (Figure 9).

## 3. Discussion

Tyrosinase is a key enzyme in melanogenesis, and mushroom tyrosinase is generally used for screening melanogenesis inhibitors. This is because the mushroom tyrosinase from *Agaricus bisporus* is commercially available and less costly compared to other sources. However, mushroom tyrosinase is significantly different from human tyrosinase in terms of substrate specificity and activity [47]. Mushroom tyrosinase is a tetramer enzyme present in the cell cytosol, whereas human tyrosinase is a monomeric, transmembrane protein located in melanosomes. In addition, the similarity of amino acid sequence between human and mushroom tyrosinase is 23% [7]. Another study has also reported on the molecular motifs’ distinctively different requirement of human tyrosinase inhibitor compared to the mushroom tyrosinase [47]. Thus, it is necessary to confirm the effectiveness of new tyrosinase inhibitors in cell culture. 

The murine melanoma cell (B16F10) is widely used to evaluate the cytotoxic effect, melanin production, and antimelanogenic effect of test materials, because they are relatively easy to culture in vitro, and they share most of the melanogenic mechanisms of normal human melanocytes [48,49]. Kojic acid and arbutin are commonly used as positive control for tyrosinase inhibitor studies [11,50]. To ensure the cytotoxic effect of hdTIPs and positive controls (arbutin and kojic acid), the MTT assay on B16F10 was performed. Our results were comparable to other studies indicating the non-toxic effect and consistent anti-tyrosinase activities of KA and arbutin on melanoma cells [51,52]. The cell viability of melanoma after treatment with hdTIPs at maximum concentration of 200 μM remained at a high survival rate (more than 90%) after 24 h (Figure 1B), 48 h and 72 h (Table S2).

In this study, to develop a safe and effective tyrosinase inhibitor, eight hdTIP candidates were selected from the machine learning-based anti-tyrosinase prediction. KNN1 showed outstanding inhibitory potency against mushroom tyrosinase (*IC*_50_ = 70.83 μM), which is similar to the *IC*_50_ of kojic acid but did not inhibit either cellular tyrosinase or melanin content in B16F10. This result might indicate that the inhibition rates of mushroom tyrosinase did not represent the melanogenesis inhibition rates in cells. According to Kim, et al. [53], oxyresveratrol and mulberroside A showed almost the same inhibition of cellular tyrosinase and melanin synthesis, but oxyresveratrol showed stronger inhibition of mushroom tyrosinase than mulberroside A. Similarly, Ochaiai et al. (2016) demonstrated two peptides that significantly inhibit the mushroom tyrosinase but did not inhibit melanin production in melanoma cells, suggesting they do not inhibit the mammalian tyrosinase [10]. In contrast, RF1 showed the best inhibition on cellular tyrosinase (18.26%) and melanin content (10.69%) in UV-induced melanoma cells at 70 μM without any cytotoxicity. Nevertheless, RF1 did not inhibit mushroom tyrosinase, indicating that the peptide did not affect anti-melanogenesis via inhibiting tyrosinase activity directly. Moreover, it probably downregulated tyrosinase expression but had no effect on tyrosinase catalytic activity. Furthermore, Qiao, et al. [54] show that *G. hederacea* extract does not inhibit mushroom tyrosinase activity but does inhibit cellular tyrosinase activity in a dose-dependent manner. 

According to the predicted antioxidative scores by AnOxPePred, TIP2 and KNN1 were putative TIPs with the highest predictive antioxidative scores in free radical scavenging and ion chelating properties, respectively. Interestingly, TIP1, TIP2, RF1, and KNN1 were listed as the top three antioxidant peptide candidates of both properties. There were several reports on the correlation between antioxidant and anti-tyrosinase properties as the high anti-oxidative peptides also showed strong anti-tyrosinase activity [23,55,56,57]. Therefore, the reduction in melanin content of melanoma cells after treatment with TIP2 and RF1 could be the combined effects from antioxidative, anti-tyrosinase, and ROS reduction mechanisms. For particular anti-oxidation, TIPs can decrease the ROS level catalyzed by tyrosinase or activate an enzyme system, including super-oxide dismutase (SOD), catalase (CAT) and glutathione peroxidase (GPx), to scavenge free radicals that stimulate tyrosinase [23]. This similar effect on oxidative stress reduction through antioxidative enzymes can also be found in phenolic compounds (phenolic acids and flavonoids) from fruit wines [58]. 

The polyphenol oxidase subunit of tyrosinase from *Agaricus bisporus* (2Y9X) contains four chains (A, B, C, D), while the other chains (E, F, G, H) belong to the subunit of lectin-like fold protein. The light subunit or lectin-like domain of mushroom tyrosinase is a protein with unknown function and not directly involved in the inhibiting process of tyrosinase inhibitors [59,60]. Since the preferred orientation of tyrosinase inhibitor and TIPs is on the polyphenol oxidase subunit, the lectin-like subunit is usually removed before molecular docking simulation [61,62]. In this study, chain D of polyphenol oxidase was selected to investigate the binding conformation to hdTIPs due to the best binding energy of TIPs-subunit chain complex obtained from the GOLD-docking results in the previous study. The molecular docking was first validated by re-docking the crystal ligand to ensure that the molecular docking could recapture. Four chains of 2Y9X docking to ligand OTR were compared and the one with the best binding score was selected for docking with hdTIPs. The results demonstrated that chain D provided the best docking orientation and conformation compared to the other chains of polyphenol oxidase subunit.

The process of bioinformatics prediction and screening for the tyrosinase inhibitory peptides is quite challenging due to the limitations of the specific in silico screening tools available. Currently, several bioinformatic pipelines and in silico screening are becoming alternative preliminary approaches for the discovery and development of the bioactive compounds or drugs and have the benefits of cutting costs and speeding up the process before the validation by in vitro and in vivo experiments [63,64,65,66,67,68]. Since there was no “ideal perfect docking program” that could give the highest accuracy and best performance in all cases, both docking approaches were employed to obtain the comparative binding energies and affinities. According to the comparative study of 14 docking programs on protein–peptide complexes by Weng et al. [69], GalaxyPepDock (as a template-based docking approach) performs the best compared to other template-based docking programs and significantly better than any template-free docking programs. HPEPDOCK, on the other hand, performs the best and is more computationally efficient for global docking compared to other programs. In this study, both programs were chosen to carry out the molecular docking in order to assess and rank the binding energy and affinity of all TIP candidates on tyrosinase. According to our docking simulation, the hydrogen bond distance ranged from 2.7 to 3.7 Å in GalaxyPepDock and 1.9 to 4.0 Å in HPEPDOCK results. Jeffrey [70] categorizes hydrogen bonds with donor-acceptor distances of 2.2–2.5 Å as “strong, mostly covalent”, 2.5–3.2 Å as “moderate, mostly electrostatic”, and 3.2–4.0 Å as “weak, electrostatic”. The proper distance of the hydrogen bond among donor-acceptor pairs is within 2.7 to 3.3 Å, with the common value as 3 Å [71]. These data demonstrated that our peptides have a moderate and weak covalent bond with the tyrosinase. According to the global docking result between hdTIPs and mushroom tyrosinase (2Y9X), KNN1 showed the most similar binding affinity of (−9.8 kcal/mol) compared to the best-known peptide, P4 (−9.3 kcal/mol). This has also been confirmed by our in vitro experiment result that KNN1 had the highest performance (*IC*_50_ = 70.83 μM) on mushroom tyrosinase inhibition compared to other hdTIP candidates. Focusing on the molecular docking scores, KNN1 had a much stronger molecular docking score (−47.9085 kJ/mol) than the peptide P4 (−19.5013 kJ/mol). Interestingly, the correlated pattern on the binding energy ratio between KNN1 and P4 was observed. Since the low van der Waals (VDW) energy ratio compared to other type of energies has been estimated, hydrogen bond and electrostatic energy seem to be the major binding energy for TIPs on mushroom tyrosinase (Table 2). This similar evidence was also been observed by the result from RING analysis, six types of interaction defining contacts based on a distance cutoff (Table S4). Even though KNN2 showed best in binding affinity (−12.5 kcal/mol) and RF2 showed the best molecular docking score (−225.6113 kJ/mol) among all hdTIPs, their major binding energies were greatly affected by VDW energy (almost 70%). Notably, KNN2 and RF2 did not show any inhibition on diphenolase activity of mushroom tyrosinase. Therefore, this pattern of binding energy ratio should also be considered as a guideline for TIPs screening. For the molecular dynamic simulation, the analysis of root-mean-square deviation (RMSD) profile is crucial to define the compactness of proteins after the ligand-induced fit into the protein complex [72,73]. All molecular docking results and molecular dynamics profiles demonstrated the prolonged and robust binding of hdTIPs to the target tyrosinase and involvement of potential binding energies with the correlation of molecular dynamics profiling and the stability of the tyrosinase−hdTIPs complexes.

In summary, this study reveals the anti-tyrosinase properties of *Haliotis diversicolor* tyrosinase inhibitory peptides (hdTIPs) based on the in vitro experiments, i.e., mushroom tyrosinase, cellular tyrosinase, and melanin content assays. The in silico validation was also performed to ensure the binding conformation between peptides and tyrosinase by molecular docking and dynamics study. All the hdTIPs are probable non-toxic to the cells up to the highest concentration of 200 μM. Among these peptide candidates, KNN1 showed the highest potent inhibitory effect on mushroom tyrosinase with an *IC*_50_ of 70.83 μM, which was close to a well-known whitening agent, kojic acid (*IC*_50_ = 61.65 μM). This high tyrosinase inhibition efficacy is also correlated with the high binding affinity, binding energy, and stability, confirmed by the molecular docking and dynamics simulations. At the concentration of 70 μM, RF1 shows the greatest inhibitory effect on cellular tyrosinase and melanin content, with 18.26 ± 1.46% and 10.69 ± 0.48%, respectively. Accordingly, it can be assumed that our hdTIPs, especially KNN1 and RF1, exhibit high potential in medical cosmetology application. In the future, the in vivo experiment or 3D skin model investigations can be further employed to ensure the actual skin effect (anti-melanogenic activity with non-cytotoxic and non-allergenic side-effects) of peptides for cosmetic and pharmaceutical applications.

## 4. Materials and Methods

### 4.1. Biomimetic Synthetic Peptide

hdTIPs, eight candidates from the TIP predictors, were chemically synthesized by Cellmano Biotech Limited (Hefei, China). The purity of the synthesized peptides was in the range of 98.18–99.80%.

### 4.2. Melanoma Cell Culture

B16F10 melanoma cells (CRL-6475™; ATCC, USA) at a passage number of P12 were cultured in Dulbecco’s modified Eagle’s medium (DMEM; ATCC, Manassas, USA) high glucose supplemented with 10% heat-inactivated fetal bovine serum (FBS; Gibco, New York, USA), 1% penicillin/streptomycin in a humidified atmosphere containing 5% CO_2_ at 37 °C. The culture medium was changed every two days [74].

### 4.3. Cell Viability Assay (MTT Assay)

A cell viability was determined using MTT assay to estimate the probable cytotoxic effect of hdTIPs and positive controls (kojic acid and arbutin) on melanoma cells. The number of viable cells was determined by the ability of mitochondria to convert MTT to formazan dye. The quantity of formazan formed is proportional to the number of viable cells present and can be measured spectrophotometrically. The method for MTT assay in this study was modified from Zaidi et al. [74]. Briefly, when cells density from Section 4.2 reached 70% of culture flask, the remaining adherent cells were trypsinized for 5 min, counted by a hemocytometer and seeded into 96-well plates at 10 × 10^4^ cells/mL, then incubated overnight. The cells were then treated with each hdTIP candidates (TIP1, TIP2, KNN1, KNN2, KNN3, RF1, RF2, and RF3) at various concentrations of 25, 50, 100, 200 μM, along with the two positive controls, kojic acid (KA) and arbutin at various concentrations (0, 125, 150, 500, and 1,000 μg/mL). After 24 h incubation, 1 mg/mL of MTT (Invitrogen, Eugene, USA) solution was replaced prior to incubation at 37 °C for 3 h. The formazan precipitates were dissolved by 100 μL of dimethyl sulfoxide (DMSO) and the concentrations were measured at 570 nm in a microplate reader (Synergy H1, BioTek, Santa Clara USA). Cell viability was calculated using the following formula: cell viability (%) = (*A*_sample_/*A*_control_) × 100, where *A*_sample_ and *A*_control_ are the absorbances from the mixture with or without the addition of test sample, respectively.

### 4.4. Mushroom Tyrosinase Activity Assay

The inhibitory effect on mushroom tyrosinase was determined spectrophotometrically via L-DOPA oxidation. The reaction mixture consisted of 20 μL of hdTIPs, 140 μL of 100 mM sodium phosphate buffer (pH6.8) and 40 μL of 200 units/mL mushroom tyrosinase (EC 1.14.18.1; Sigma, Saint Louis, USA) placed in a 96-well microplate, then incubated for 10 min. After that, 20 μL of 5mM L-DOPA were mixed into each well over 20 min. Furthermore, the amount of dopachrome produced absorbance was measured at 475 nm. In addition, KA was used as the positive control at the same concentrations and conditions to hdTIPs. This method was modified from Kim et al. [53].

The mushroom tyrosinase inhibitory activity was calculated with the following equation:

$$\%\;Mushroom\;tyrosinase\;inhibition=\left[\frac{\left(C\right)-\left(S\right)}{\left(C\right)}\right]\times 100,$$

where, *C* represents the OD_475_ of control and *S* is the OD_475_ of sample. All experiments were performed in triplicate to determine the *IC*_50_ of the samples.

### 4.5. Intracellular Tyrosinase Activity Assay

Tyrosinase activity was measured based on Dopa oxidase activity by estimating the dopachrome production according to the method by Qiao et al., 2012 [54], with some modifications. B16F10 cells were seeded at 5 × 10^6^ cells/mL in 24-well plates. Cells were incubated for 24 h with 70 μM of hdTIPs and 100 μg/mL of kojic acid or arbutin as positive controls. Each well was then washed and replaced with PBS 600 μL and induced with UVA (314–400 nm, 3.0 W) and UVB (280–315 nm, 13.6 W) using OSRAM (Ultra-Vitalux, Germany) for 22 s (0.32 J/cm^2^). After that, the cells were incubated with medium 600 μL/well for a further 24 h. Then, the cells were trypsinized and the harvested cells were lysed in cell extraction buffer. After normalized protein concentration, 10 μL of each lysate supernatant was aliquoted into a 96-well plate and shaking incubated in dark conditions with 1 mM L-DOPA 90 μL in PBS at 37 °C at 280 rpm for 10 min. The absorbance was measured at 475 nm and all experiments were performed in triplicate to determine the *IC*_50_ of the samples.

The cellular tyrosinase inhibitory activity was calculated with the following equation:

$$\%\;Cellular\;tyrosinase\;inhibition=\left[\frac{\left(C\right)-\left(S\right)}{\left(C\right)}\right]\times 100,$$

where, *C* are defined as in the previous section.

### 4.6. Melanin Content Assay

Cellular melanin content was measured using a modified method by Jegal, et al. [75]. B16F10 cells were seeded at 5 × 10^5^ cells/mL in 24-well plates. Cells were then incubated for 24 h with 70 μM hdTIPs and 100 μg/mL kojic acid or arbutin as positive controls. After that, the cells in each well were washed and replaced by PBS. The UV stimulation process was performed by the exposure to UVA (314–400 nm, 3.0 W) and UVB (280–315 nm, 13.6 W) for 22 s. Then, PBS was replaced with media and further incubated for 24 h. The UV-stimulated cells were then trypsinized and centrifuged at 1500 rpm for 5 min. The cell pellet was then dissolved in 2 M NaOH at 60 °C for 1 h to solubilize the melanin. The melanin content was estimated by measuring the absorbance at 405 nm.

The melanin content was calculated with the following equation:

$$\%\;Melanin\;content=\frac{\left(S\right)}{\left(C\right)}\times 100,$$

where, *S* is the OD_405_ of sample and *C* represents the OD_405_ of control. All experiments were performed in triplicate.

### 4.7. Assay of Antioxidative Enzymes Activities

To determine the antioxidant activity, the centrifugation precipitate from Section 4.6 was dissolved in 1 mL of 1 M NaOH containing 10% DMSO at 80 °C for 1 h. Various assay kits, all from Beijing Solarbio Science & Technology Co., Ltd., Beijing, China, were used to measure the quantity of reduced reactive oxygen species (ROS) and the activity of superoxide dismutase (SOD), catalase (CAT), and glutathione peroxidase (GPx) according to the methods by Hu et al. [22].

### 4.8. Antioxidant Peptides Prediction and Molecular Docking Simulation

Peptide sequences of all selected TIPs were submitted to the AnOxPePred web server (https://services.healthtech.dtu.dk/service.php?AnOxPePred-1.0) [76] (accessed on 23 December 2022) to predict free radical scavenging and ion chelating activities. The peptide mode was selected as analyze mode, and minimum and maximum peptide length were set as 7 and 13 amino acids, respectively.

A protein–peptide docking web server based on interaction similarity and energy optimization “GalaxyPepDock” via http://galaxy.seoklab.org/pepdock/ (accessed on 20 July 2022) and a web server for blind protein-peptide docking based on a hierarchical algorithm “HPEPDOCK” via http://huanglab.phys.hust.edu.cn/hpepdock/ (accessed on 21 July 2022) were used to study the conformation and binding energy of hdTIPs with tyrosinase. P4, the best-known model peptide with high potential on inhibit mushroom tyrosinase according to Ochiai et al. [44], was used as positive control. To prepare the tyrosinase structure, the crystal structure of tyrosinase from *Agaricus bisporus* was downloaded from PDB: 2Y9X [77]. According to our previous work, the preferred orientation of TIPs are only on the polyphenol oxidase, with tetramers A, B, C, and D chains [42]. Therefore, all the chains of lectin-like subunit (E-H) were removed by Chimera. The ligands on polyphenol oxidase, ion and water molecules were removed using BIOVIA Discovery Studio visualizer 21.1.0.0. The best ten models were then visualized using UCSF Chimera 1.13.1 to define the hydrogen bond [78]. PRODIGY server via https://wenmr.science.uu.nl/prodigy/ (accessed on 23 July 2022) and PIMA server via http://caps.ncbs.res.in/pima/ (accessed on 23 July 2022) were used to predict binding affinity and other interactions.

### 4.9. Molecular Dynamics Simulation

The Amber ff14DB force field and Amber16 software package [79] were used to perform MD simulations of four systems (tyrosinase-hdTIPs complexes). The TIP3P water model was used to solvate each system at a distance of 10 Å from the protein. The sodium ions were added to neutralize the simulated systems. For KNN1, RF1, RF3, and TIP2, the simulation box dimensions are 79 × 82 × 89 Å^3^, 80 × 82 × 89 Å^3^, 82 × 82 × 89 Å^3^, and 82 × 77 × 78 Å^3^, respectively. The protein-ligand complexes (KNN1, RF1, RF3, and TIP2) have 398, 398, 401, and 404 residues, respectively. The entire system was solvated at distance of 10 Å from the protein surface. The system approximate atom of TIP3P water models for KNN1, RF1, RF3, and TIP2 were 45,351, 44,568, 47,115, and 34,113 atoms, respectively. The initial conformations were heated to 300 K with a canonical ensemble (NVT) for 100 ps before being equilibrated for another 1200 ps. Then, until 300 ns of the production run, all-atom MD simulations were performed under the isothermal-isobaric ensemble (NPT) at 1 atm and 300 K with a simulation time step of 2 fs. The Berendsen barostat [80] with a pressure-relaxation time of 1 ps and the Langevin thermostat [81] with a collision frequency of 2 ps-1 were used to maintain pressure and temperature during MD simulation, respectively. The SHAKE algorithm [80] was used to constrain all chemical bonds involving hydrogen atoms, while the particle mesh Ewald’s (PME) summation method [82] was used for the treatment of the long-range electrostatic interactions. The cut-off for non-bonded interactions was set at 10 Å. The CPPTRAJ module of AMBER16 was used to calculate particular parameters in structural analysis such as root-mean-square deviation (RMSD), root-mean-square fluctuation (RMSF), radius of gyration (Rg), and hydrogen bond profile. These parameters were investigated during the simulation period of 300 ns. To look at time-dependent properties of all possible protein-ligand interactions, the RING analysis was performed by RING 2.0 server (https://ring.biocomputingup.it) [83] (accessed on 23 January 2023). Numerous intra-protein interactions in complex systems, such as π-π stacking, ionic bonding, hydrogen bonding, and van der Waals interactions, were observed. Ten models from 300 ns-simulated time (collected once every 30 ns) were exact to analysis.

## 5. Conclusions

In silico prediction tools are useful for studying tyrosinase inhibitors, being a time- and cost-effective alternative. The in vitro assay demonstrates KNN1 decreases 50% of mushroom tyrosinase activity at concentration 70.83 μM, while kojic acid *IC*_50_ = 61.65 μM. In addition, RF1 at 70 μM can inhibit almost 18.26% and 10.69% of cellular tyrosinase and melanin content, respectively. Furthermore, none of the abalone biomimetic peptides showed a significant cytotoxic effect to the murine melanoma cells. Thus, these bioactive peptide candidates were promising as safe and effective tyrosinase inhibitors in the development of melanin-reducing agents. However, further investigation in in vivo or 3D skin models are still needed to ensure the actual skin effect of peptides for cosmetic and pharmaceutical applications.

## Figures and Tables

**Table 1:** Abalone predicted TIPs by KNN and RF-based predictors and the predicted antioxidant scores by AnOxPePred 1.0 program.

hdTIPs	Peptide Sequences	Predicted Anti-Tyrosinase Probability		Predicted Antioxidant Scores	
		KNN Predictor	RF Predictor	Free Radical Scavenging (Rank)	Ion Chelating (Rank)
TIP1	TASSDAWYR	0.97	0.71	0.37 (3)	0.21 (2)
TIP2	SAPFMPDAFFRNV	0.79	0.54	0.44 (1)	0.21 (2)
KNN1	NICECMK	1.00	0.39	0.37 (3)	0.25 (1)
KNN2	TSQMSRSSSR	1.00	0.37	0.27 (5)	0.21 (2)
KNN3	KKNYRVSEAYK	1.00	0.32	0.25 (6)	0.19 (4)
RF1	SAPTFFR	0.00	0.63	0.39 (2)	0.21 (2)
RF2	NSSLRVQSR	0.00	0.60	0.24 (7)	0.20 (3)
RF3	SQSNSRSVSR	0.00	0.52	0.34 (4)	0.15 (5)

**Table 2:** Calculated binding affinity (∆G), dissociation constant (Kd), and binding energy scores from the molecular docking results from HPEPDOCK of peptides to the tyrosinase based on the PROGIDY and PIMA web servers.

Protein-Peptide Complex	∆G (kcal/mol)	Kd (M) at 25.0 °C	H-Bond Ener. (kJ/mol)	Elec. Ener. (kJ/mol)	VDW. Ener. (kJ/mol)	Molecular Docking Score (kJ/mol)
2Y9X—TIP1	−9.8	6.4 × 10^−8^	−16.5114	26.7705	−102.934	−92.6749
2Y9X—TIP2	−8.5	5.4 × 10^−7^	−38.7443	0	68.545	29.8007
2Y9X—KNN1	−9.8	6.8 × 10^−8^	−11.9095	−40.3499	4.3536	−47.9058
2Y9X—KNN2	−12.5	7.1 × 10^−10^	−29.1007	−32.8336	−133.06	−194.9942
2Y9X—KNN3	−10.3	2.9 × 10^−8^	−27.1574	−17.0376	−144.366	−188.561
2Y9X—RF1	−8.5	6.3 × 10^−7^	−27.8065	−4.7535	−117.47	−150.03
2Y9X—RF2	−9.3	1.6 × 10^−7^	−37.0432	−35.4341	−153.134	−225.6113
2Y9X—RF3	−10.6	1.6 × 10^−8^	−31.213	−21.6992	−104.05	−156.9622
2Y9X—P4	−9.3	1.6 × 10^−7^	−34.23	24.0025	−9.2737	−19.5013

## Data Availability

Not applicable.

## Supplementary Materials

The following supporting information can be downloaded at: https://www.mdpi.com/article/10.3390/ijms24043154/s1.
